# Nascent chain folding feeds back on mRNA stability through the ribosome: Consequences for therapeutic mRNA design

**DOI:** 10.1016/j.omtn.2026.103070

**Published:** 2026-09-10

**Authors:** Suhee Chang, Jeff Coller

**Affiliations:** 1 Department of Molecular Biology and Genetics, Johns Hopkins University School of Medicine, Baltimore, MD, USA

## Main text

**Figure 1:**
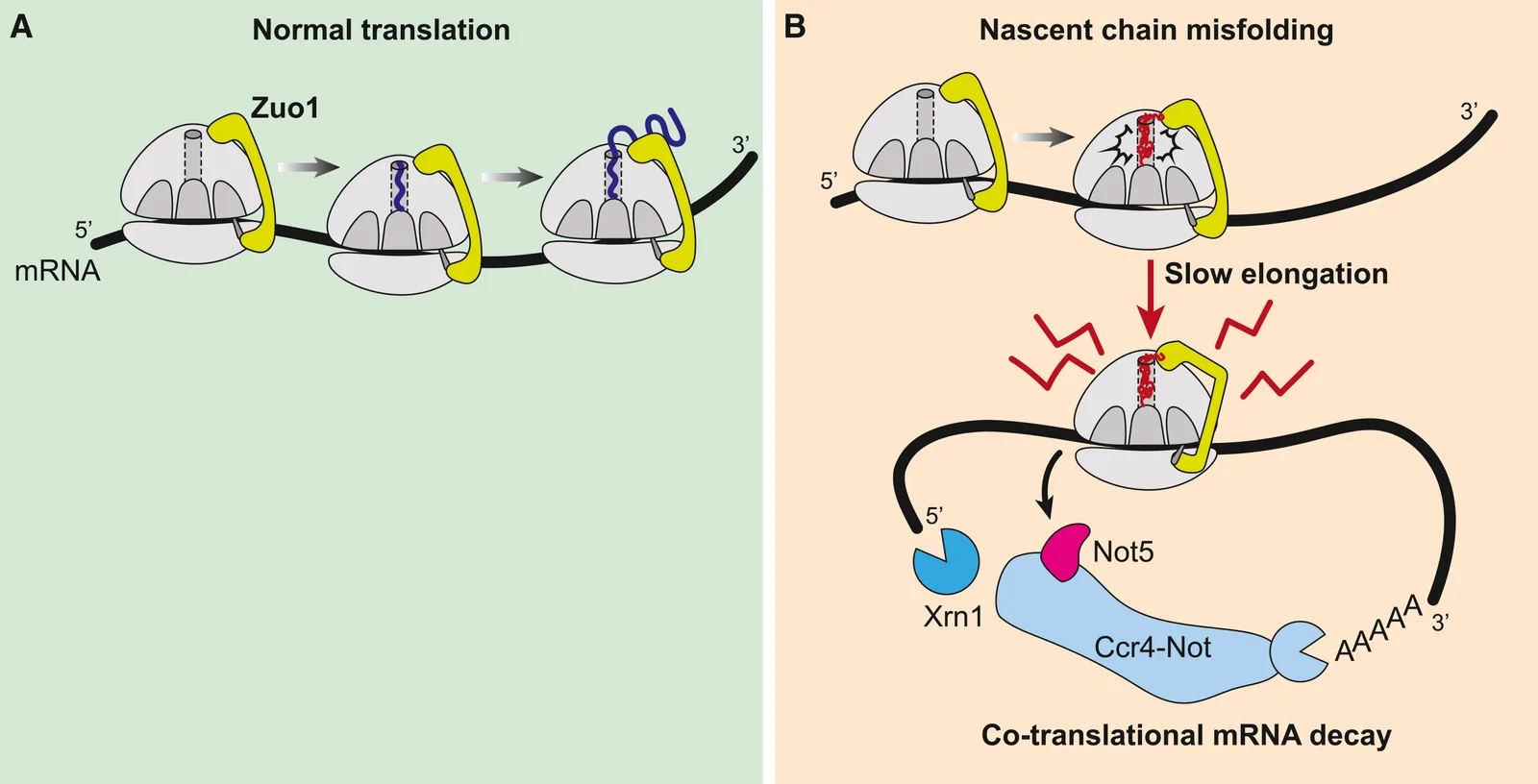
Schematic of Zuo1-mediated ribosome regulation in response to nascent chain misfolding (A) During normal mRNA translation, Zuo1 (lime) bridges both ribosomal subunits, positioning near the ribosomal exit channel (top circle) where the nascent chain (blue) emerges, while also interacting with H44 (gray rod), which extends from the ribosomal A-site. (B) Upon nascent chain misfolding (red), Zuo1 slows the elongating ribosome. The slowed ribosome is recognized by Not5 (pink), which recruits Xrn1 (cyan) and the Ccr4-Not complex (sky blue) to initiate co-translational decay of the transcript.

In our recently published study in *Molecular Cell*,^1^ we describe a surveillance mechanism in which misfolding of the nascent chain slows the elongating ribosome and triggers co-translational decay of the mRNA encoding it (Figure 1). We propose that the ribosome-associated chaperone Zuotin (Zuo1) mediates this feedback, coupling nascent chain folding to ribosome dynamics and mRNA stability. The finding bears directly on how therapeutic mRNAs are designed, because the codon optimization strategies used to accelerate elongation and stabilize transcripts may compromise co-translational folding and engage the very pathway that destabilizes them.

During translation, ribosome dynamics along a transcript determine mRNA fate: abnormal ribosome movements are detected by mRNA decay machineries that trigger co-translational degradation of the transcript.^2^ Codon usage and tRNA availability are well-known determinants of elongation rate. However, whether the protein product itself, which remains physically tethered to the ribosome throughout translation, feeds back on ribosome regulation has not been widely investigated.

We focused on a key feature of nascent chains: their folding status. Nascent chains begin folding co-translationally, and ribosome dynamics play an important role in proper folding of the protein product.^3^ While the role of ribosome speed is established in protein folding, whether feedback exists from the nascent chain to the ribosome, and how such a signal could be transmitted, remained unclear. The nascent chain emerges from the exit tunnel of the large subunit, physically distant from the ribosomal A-site where decoding occurs. Therefore, in order for the nascent chain to regulate ribosome decoding rate, communication between these sites requires a mediator. Zuo1, a component of the ribosome-associated complex (RAC), is uniquely positioned for this role^4^: its N-terminal region interacts with emerging nascent chains at the ribosomal exit, while its C-terminal region contacts ribosomal RNA helix 44, which extends from the decoding center. We hypothesized that Zuo1 mediates communication between nascent protein folding and ribosome speed, and through that, mRNA stability.

To test whether nascent protein folding affects mRNA stability, we first examined transcriptome-wide changes induced by global protein misfolding stress. Under three different conditions that induce global protein misfolding (heat, oxidative stress, and treatment with the proline analog AZC), Zuo1 was required for the transcriptomic changes beyond the canonical heat-shock response. To directly assess the role of protein misfolding in mRNA stability, we generated reporter constructs by codon-shuffling the endogenous yeast *HIS3* gene. These reporters maintain the identical codon composition of *HIS3*, but because the codon order is scrambled, the encoded protein is largely unstructured. Unlike the endogenous *HIS3* mRNA, the shuffled transcripts were destabilized in a Zuo1-dependent manner, demonstrating that Zuo1 is required for selective destabilization of transcripts encoding proteins that fail to achieve proper structure.

We provide evidence that this destabilization involves co-translational decay machinery including Not5. The shuffled reporter transcript generated a 3′ decay fragment that disappeared in cells lacking Xrn1, the 5′-to-3′ exonuclease. This indicates that a stalled ribosome on the shuffled reporter mRNA blocked Xrn1 progression, leaving a protected 3′ fragment. Indeed, the decay fragment was ribosome-bound and dependent on Not5, which detects slowed ribosomes and recruits the co-translational mRNA decay machinery. Critically, the fragment was also dependent on Zuo1, suggesting that Zuo1 mediates ribosome stalling on this reporter transcript. The Not5 dependence places this surveillance within the Ccr4-Not arm of co-translational decay, which responds to slowed elongation, and distinguishes it from ribosome quality control, where terminally stalled ribosomes are resolved through Hel2-dependent ubiquitylation and degradation of the nascent chain.

To directly measure ribosome slowdown upon protein misfolding, we pulse-labeled yeast cells with ^35^S-methionine/cysteine during AZC treatment and compared total ^35^S incorporation with the release of ^35^S-labeled protein products from ribosomes. Global protein misfolding slowed release of the protein products from the ribosome in a Zuo1-dependent manner, suggesting that Zuo1 is required for the ribosome slowdown in response to protein misfolding. Ribosome profiling, which sequences ribosome-protected mRNA fragments to map ribosome positions, confirmed that under global protein misfolding conditions, ribosomes are stalled in the early open reading frames (ORFs). This stalling was again Zuo1 dependent, highlighting Zuo1’s role in regulating ribosome movements under protein misfolding. Transcripts showing this Zuo1-dependent stalling were reduced in abundance under protein misfolding conditions, tying ribosome stalling to transcript level.

Zuo1’s key domains are well conserved in its human homolog DNAJC2 (MPP11), and we present preliminary evidence suggesting that this regulatory mechanism is conserved in human cells. Analysis of a published dataset from HEK293 cells treated with thapsigargin,^5^ which induces misfolding of ER-targeted proteins, revealed that thapsigargin treatment increased ribosome occupancy in early ORFs, and that this increase was abolished upon reduction of *DNAJC2*, mirroring our observations in yeast. Furthermore, using the cystic fibrosis transmembrane conductance regulator (CFTR), whose well-known ΔF508 variant misfolds co-translationally,^6^ we found that *DNAJC2* knockdown significantly increased steady-state levels of the ΔF508 *CFTR* mRNA without affecting wild-type *CFTR*. These data suggest that a Zuo1/DNAJC2-mediated surveillance pathway acting on transcripts encoding misfolded proteins is conserved in humans.

Together, these findings identify a layer of translational control in which the folding status of the nascent chain feeds back on ribosome dynamics and mRNA fate. The broader implication is that the protein product of a transcript helps set the stability of that transcript. This matters for nucleic acid therapeutics. Accelerating elongation is a commonly used strategy to stabilize mRNA, but faster elongation can compromise co-translational folding, potentially triggering Zuo1-mediated surveillance and destabilizing the very transcript the optimization was meant to protect.

Two future goals emerge from this work. First, identifying which folding defects are recognized and when Zuo1 is recruited to elongating ribosomes will be essential for designing therapeutic mRNAs that avoid triggering this pathway. Second, elucidating the mechanism by which Zuo1 regulates ribosome dynamics remains an open question. Zuo1 adopts two conformations on the ribosome, switching the ribosomal proteins it contacts and bending helix 44 to different angles: how this conformational switch relates to ribosome regulation is unknown. A mechanistic answer may open this pathway to fine-tuning in the design of mRNA-based medicines.

## Acknowledgments

This work was supported by 10.13039/100015283Bloomberg Philanthropies, 10.13039/100000897Cystic Fibrosis Foundation (006606F223) and the 10.13039/100000002National Institutes of Health (R35GM144114).

## Declaration of interests

The authors declare no competing interests.
